# Epidemiology of Visceral Leishmaniasis with Emphasis on the Dynamic Activity of Sand Flies in an Important Endemic Focus of Disease in Northwestern Iran

**DOI:** 10.18502/jad.v14i1.2716

**Published:** 2020-03-31

**Authors:** Ehssan Mozaffari, Hassan Vatandoost, Yavar Rassi, Mehdi Mohebali, Amir Ahmad Akhavan, Eslam Moradi-Asl, Zabihola Zarei, Alieza Zahrai-Ramazani, Esmail Ghorbani

**Affiliations:** 1 Department of Medical Entomology and Vector Control, School of Public Health, Tehran University of Medical Sciences, Tehran, Iran; 2 Department of Environmental Chemical Pollutants and Pesticides, Institute for Environmental Research, Tehran University of Medical Sciences, Tehran, Iran; 3 Department of Parasitology and Mycology, School of Public Health, Tehran University of Medical Sciences, Tehran, Iran; 4 School of Public Health, Ardabil University of Medical Sciences, Ardabil, Iran

**Keywords:** *Phlebotomus kandelakii*, *Phlebotomus perfilewi transcaucasicus*, Visceral leishmaniasis, Iran

## Abstract

**Background:**

Leishmaniasis diseases are known to be one of the most important public health problems in World and Iran. Visceral leishmaniasis is considered to be the most serious form and transmitted by sand flies species. The aim of this study was to investigate the dynamic activities of sandflies in northwestern Iran.

**Methods:**

This crass-sectional study was conducted from April to December 2018 in Meshkinshahr County, Ardabil Province. Sticky traps have been used to collect sand flies. They are stored in 70% alcohol and finally identified using valid keys. The aspects of the synoptic information were inquired from the Meshkin Shahr weather department and results analyzed for SPSS24.

**Results:**

Totally 259 sandflies were collected during study period. From collected samples 78.7% were male and 21.3% female. There were 8 different species. *Phlebotomus kandelakii* was the most prevalent one (30.8%). The average temperature, relative humidity, and average wind speed was 13.5 °C, 84%, and 2 meters per hour on the onset of sandflies’ activity, respectively. These values were 18.3 °C, 85% and 1.5 meters per hour at the peak of their activity and 16 °C, 62% and 5 meters per hour at the final stage of their activity. Sand flies had one peak in July which is strongly influenced by temperature and humidity conditions. Two species of *Ph. kandelakii* and *Phlebotomus perfilewi transcaucasicus*, had the highest activity in this endemic area.

**Conclusion:**

The results of current study will provide a guideline for control of diseases in the country.

## Introduction

Arthropod-borne diseases are known to be one of the most important public health problems. Today, more than one third of infections are the direct result of arthropod-borne diseases (1). Leishmaniasis is a complex disease which is transmitted by vectors that carrying more than 20 different types of Leishmania parasites belonging to the Kinetoplastida class (2, 3). Leishmaniasis has been reported in more than 101 countries worldwide (4), with more than 350 million people living in risky areas around the world (2, 5). The most important vectors of leishmaniasis were Phlebotomus sandflies in the past and are currently Lutzomyia sandflies (6). In terms of clinical symptoms, leishmaniasis infection can be classified as cutaneous leishmaniasis (CL), visceral leishmaniasis (VL) and mucocutaneous leishmaniasis (MCL) in which visceral leishmaniasis (also called kala-azar) is considered to be the most serious form (7). Visceral leishmaniasis affects between 0.2 and 0.4 million people and causes approximately 40,000 deaths each year (6). Visceral leishmaniasis in Iran belongs to the Mediterranean type with the *Leishmania infantum* agent. Dogs and canines have been identified as the main reservoirs of the disease and approximately 100–300 human cases are registered every year in Iran (8, 9). To date, a total of 44 sandflies species have been identified, and 10 more species are still under investigation (10). The most recent study in the endemic focus of the province of Ardabil has described 22 species of sandflies dispersed throughout the region (11). Definitive vectors of visceral leishmaniasis in the province of Ardabil belong to the larrossious subgenus, including the species *Phlebotomus kandelakii*, *Phlebotomus perfilewi transcaucasicus* and *Phlebotomus tobbi* (12–14). Parasitic infection has also been confirmed in three areas of the province of Ardabil: Meshgin Shahr, Germi and Bileh Savar (15). *Phlebotomus kandelakii*, the main vector of visceral leishmaniasis in northwestern Iran, is a mountainous sandfly that lives in special conditions. The study of the ecological behavior and dynamic activities of such vectors may help to prevent and control leishmaniasis diseases (16). The aim of this study is therefore to investigate the dynamic activities of sandflies with an important endemic focus in northwestern Iran.

## Materials and Methods

### Study area

**Fig. 1.**
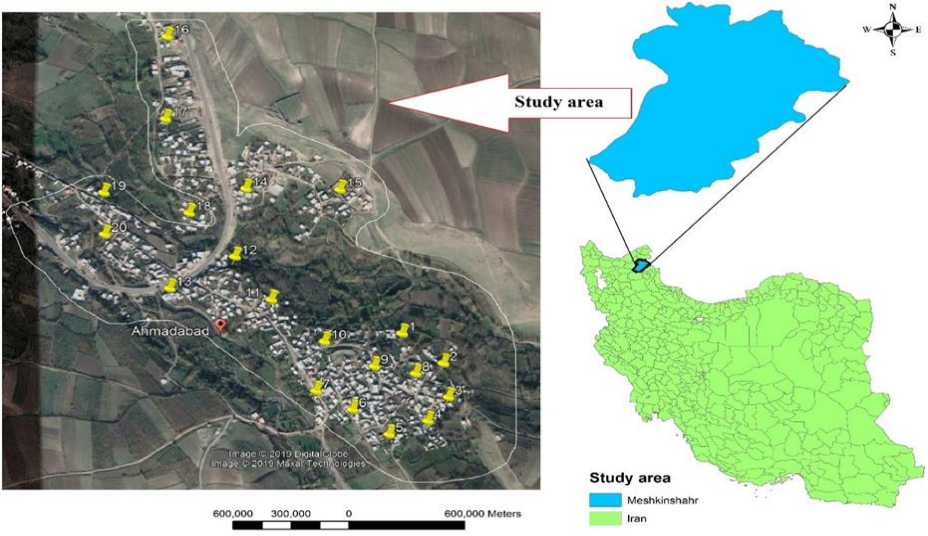
Field of research and areas where sandflies are caught in northwestern Iran

The province of Ardabil is located in the north-west of Iran. This study was conducted in the district of Meshgin Shahr, Ahmad Abad village located in the center of Ardabil *Phlebotomus*, from April to December 2018 (Fig. 1). More than 50 percent of visceral leishmaniasis cases have been identified in the selected area all through the province.

### Data collection

Information on cases of disease was obtained from a health center in Meshkin Shahr. The relevant information regarding temperature, humidity, and wind speed were measured and recorded by maximum/minimum thermometer and silver barometer on a daily basis in 5 different, fixed locations. Other aspects of the synoptic information were inquired from the Meshkin Shahr weather department. A maximum of 60 sticky traps (30 indoors and 30 outdoors) have been used to capture sand flies. Sticky traps were installed once every two weeks before the sunset and were collected before the sunrise. Trapped sandflies were removed from sticky traps by the insulin syringe and stored in 70% alcohol. They were then transferred to the laboratory, mounted with 1–2 drops of Puri's solution and finally identified using valid keys (16, 17).

## Results

**Fig. 2.**
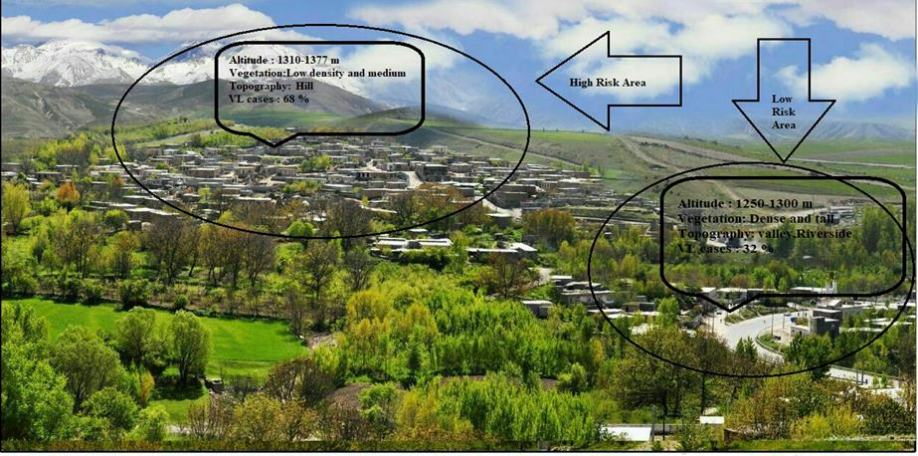
Topographic characteristics of the endemic area of visceral leishmaniasis in northwestern Iran

In this endemic focus, from 2000 to 2018, 30 disease cases were reported of which 60% were male and 30% were female. Of these 56% were less than 2 years old, 24% from 2 to 5 years old, and 20% above 5 years of age. Using the Direct Agglutination Test (DAT), the highest number of cases (32%) was found to be positive with antibody titer 1:3200 and the lowest number (4%) with antibody titer to 1:51200. The highest number of visceral leishmaniasis cases (68%) were confirmed in areas at an altitude of 1310–1380 meters and areas with average vegetation, average tree height, scarp topography, 60% rural population, farming, livestock as the main occupation, and 268 domestic dogs and flocks. On the other hand, the smallest number of visceral leishmaniasis cases have been reported from areas located along the valley topography at an altitude between 1250 and 1300 meters above sea level with dense vegetation and tall trees, 40% urban population, shop keeping as main occupation, and 98 domestic dogs and flocks (Fig. 2).

**Fig. 3.**
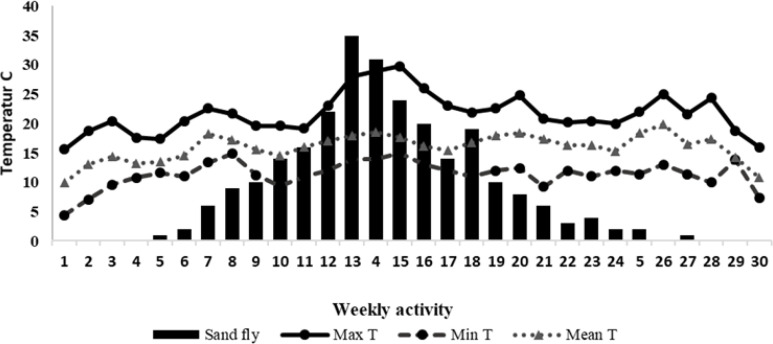
Weekly activity of sand flies based on the temperature of the region in the endemic focus of northwestern Iran

**Fig. 4.**
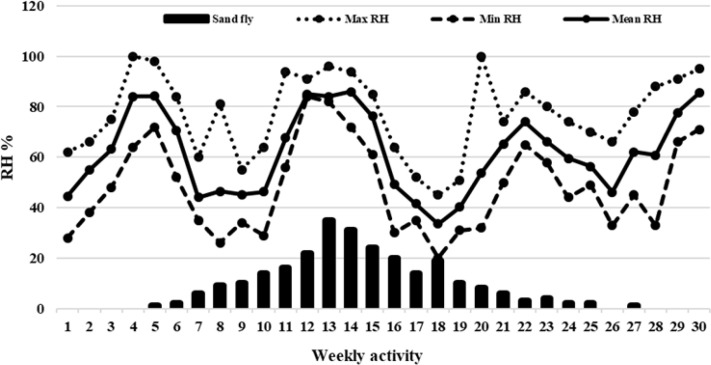
Weekly activity of sand flies based on the relative humidity of the region in the endemic focus of northwestern Iran

**Fig. 5.**
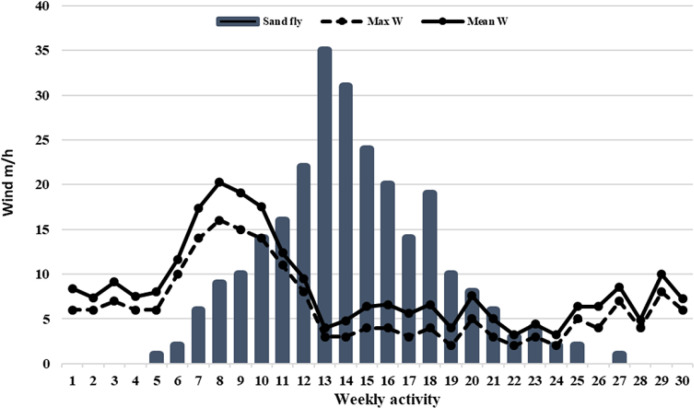
Weekly activity of sand flies based on the wind speed of the region in the endemic focus of northwestern Iran

A total of 259 sandflies were trapped by sticky traps during the 30 weeks of the study period (April to December 2018) of which 78.7 % were male and 21.3% were female. There were 8 different species identified with *Ph. kandelakii* as the most frequent one (30.8%), followed by *Phlebotomus papatasi* (28.5%) and *Ph. perfiliewi transcaucasicus* (3%). Of those sandflies, 32.8% were trapped in the house yards and 3.4% in the bathrooms. The onset of the activity and appearance of vectors in this region started from the 5^th^ week of study (May 5), the peak of activity started from the 13^th^ and 14^th^ week of study (late July), and the ending point of activity started from 26^th^ and 27^th^ weeks of the study (October). The largest numbers of sandflies were trapped in barns (33%), and the lowest number in the restrooms (3.4%). *Phlebotomus kandelakii* was trapped mostly in bedrooms and barns and *Ph. papatasi* in aviculture and barns. The average temperature, relative humidity, and average wind speed was 13.5 °C, 84%, and 2 meters per hour on the onset of sandflies’ activity, respectively (Figs. 3–5). These values were 18.3 °C, 85% and 1.5 meters per hour at the peak of their activity and 16 °C, 62% and 5 meters per hour at the final stage of their activity (Table 1). Fifty-nine per cent of sand flies are caught in high-risk disease areas and 40.2% from low-risk areas. *Phlebotomus kandelakii* was mostly (80%) trapped in high-risk areas where infection with leishmaniasis has been reported in both humans and animals. During the 12^th^ to 16^th^ weeks of study, 42.5% of the sand flies were caught when the wind speed was 3–4 meters per hour with an average speed of 1–2 meters per hour. The highest recorded temperature in this period was 25–30 °C and the lowest temperature was 12–15 °C; the temperature for suitable activity was 17–18.5 °C. In the peak of activity, the highest humidity was 85–96%, the lowest was 61–84%, and the average of sufficient humidity was 76–86% (Table 2).

**Table 1.** Frequency of captured sand flies based on location in the endemic focus of northwestern Iran

**Spices**	**Male**	**Female**	**Room**	**Bathroom**	**Toilet**	**Barn**	**Birdman**	**Depot**	**Yard**	**Total**
***Ph. kandelakii***	56	24	16	2	3	35	4	5	15	80
***Ph. perfiliewi transcaucasicus***	6	2	2	1	1	2	0	0	2	8
***Ph. longidactus***	14	0	0	0	4	5	1	3	1	14
***Ph. balcanicus***	20	0	0	0	8	9	0	2	1	20
***Ph. papatasi***	68	6	3	6	5	26	21	8	5	74
***Ph. sergenti***	15	5	0	0	0	0	12	2	6	20
***Ph. mongolensis***	25	0	0	0	3	7	2	3	10	25
***Ph. caucasicus group***	0	18	0	0	4	1	2	2	9	18
**Total**	204	55	21	9	28	85	42	25	49	259

**Table 2.** Dynamic activity of sandflies on the basis of weather variables in the endemic area of northwestern Iran

**Activity**	**No. Sand fly**	**Average relative humidity at 15 o'clock**	**Average relative humidity at 03 o'clock**	**Average wet-bulb temperature**	**Average dew point temperature**	**Total daily radiation**	**Mean Relative Humidity**	**Min Relative Humidity**	**Max Relative Humidity**	**Average Daily Cloudy**	**Mean temperature**	**Min temperature**	**Max temperature**	**Min wind speed**	**Max wind speed**	**Weekly activity**
	0	41	4.8	53	−2.3	2801	44.6	28	62	2	9.9	4.4	15.6	2.375	6	1
	0	44	8.5	63	3.9	2756	54.8	38	66	2	13.1	7.1	18.7	1.375	6	2
	0	54	10.6	68	7.3	2147	63.1	48	75	4.4	14.4	9.6	20.4	2.125	7	3

**Start**	0	88	11.6	81	10.3	1386	84	64	100	4.4	13.2	10.8	17.6	1.5	6	4
	1	87	11.9	89	10.7	1243	84.2	72	98	4.4	13.5	11.6	17.4	2	6	5

	2	62	11.5	84	9.1	1822	70.6	52	84	4.4	14.5	11	20.4	1.625	10	6
	6	38	11.5	47	5.6	2516	44.1	35	60	4.4	18.2	13.4	22.6	3.375	14	7
	9	26	10.8	46	4.8	1234	46.5	26	81	4.4	17.2	14.9	21.7	4.25	16	8
	10	48	9.5	55	3.6	1292	45.2	34	55	4.4	15.6	11.2	19.6	4.125	15	9
	14	41	8.7	64	2.7	2017	46.3	29	64	4.4	14.5	9.4	19.6	3.5	14	10
	16	63	9.6	62	6.7	1801	67.6	56	94	4.4	16	11	19.2	1.375	11	11
	22	100	10.3	100	10.2	677	85	84	91	4.4	17	12	23	1.5	8	12

**Peak**	35	84	9.6	100	9.4	1806	84	82	96	4.4	18	14	28	1	3	13
	31	98	9.4	98	8.6	1905	86	72	94	4.4	18.5	14	29	1.8	3	14

	24	61	10	83	8.1	1720	76.2	61	85	4.4	17.6	15	29.8	2.4	4	15
	20	30	4.9	64	−1.4	1452	49.2	30	64	4.4	16.2	13	26	2.6	4	16
	14	35	9	52	2.3	2160	41.6	35	52	4.4	15.5	12	23	2.6	3	17
	19	30	5.2	38	−4.2	2058	33.6	20	45	4.4	16.8	11	21.9	2.6	4	18
	10	31	10.8	51	3.9	2016	40.2	31	51	4.4	18	12	22.6	2	2	19
	8	100	9.1	50	3.6	1906	53.8	32	100	4.4	18.4	12.4	24.8	2.6	5	20
	6	74	13.4	71	10.5	1845	65.2	50	74	4.4	17.4	9.2	20.8	2	3	21
	3	86	13.5	68	11.58	1789	74	65	86	4.4	16.3	12	20.2	1.2	2	22
	4	59	12.6	80	9.82	2047	66.2	58	80	4.4	16.3	11	20.4	1.4	3	23
	2	74	11	59	7.2	2149	59.6	44	74	4.4	15.3	12	20	1.2	2	24
	2	51	13.2	70	9.3	2015	56.4	49	70	4.4	18.4	11.4	22	1.4	5	25

**End**	0	36	13	66	7.4	2049	46.2	33	66	4.4	19.9	13	25	2.4	4	26
	1	56	12.2	61	8.9	1980	62	45	78	4.4	16.4	11.4	21.6	1.57143	7	27

	0	33	12.5	86	8.8	1895	60.7	33	88	4.4	17.3	10	24.4	0.875	4	28
	0	85	12	77	10.3	1987	77.6	66	91	4.4	14.3	13.8	18.8	2	8	29
	0	71	9.5	95	8.4	2105	85.5	71	95	4.4	10.8	7.4	16	1.25	6	30

## Discussion

Visceral leishmaniasis infection cases in the endemic focus of northwestern Iran, including Ardabil Province, have started to rise since 2013 and were up to four times higher in 2018. In addition, canine visceral leishmaniasis has also risen from 4% to 38% in recent years (9, 18). Previous studies in this region have already shown that 50% of certain vectors of visceral leishmaniasis in Iran have been registered from Ardabil Province (12–14) with *Ph. kandelakii* as the most common species trapped in the city of Meshgin Shahr and in the villages of Ahmad Abad, Parikhan, Oor-Kandi and Khalaf (19, 20). The identification of sandflies’ activity areas, determination of monthly frequency, activity season, the appearance and disappearance time of main vectors, and identification of the dynamic activity of vectors in endemic foci are among the most important factors that can be taken into account for vector and diseases control in this area (21–24). The appearance of sand flies in this region started from the first half of May, the peak time of their activity was at the end of July, and the final activity period is in the second half of October. However, climate change may affect these periods from 7 to 14 days. The activity period of sand flies lasts between 150–170 days on average per year. Nonetheless, it should be noted that these indexes will vary depending on the specific topographical and climatic conditions, as in the northwesterly regions, sand flies have one generation and one peak time, whereas they have 2–3 generations and 2 peak times in the southern regions (16, 25). In most areas of the WHO EMRO countries, sand flies transmitting visceral leishmaniasis have one or two peaks of activity and start to act from early April to June (22). Most of the sand flies in the current study were caught in the outdoors and previous studies in this region and other areas showed the same findings, indicating that the vectors of visceral leishmaniasis prefer to take blood and rest outside the housing areas (11, 19, 26, 27). The sand flies activity period in the study area was between 13.5 °C and 20 °C, with a relative humidity of 80–86 percent and a wind speed of between 1.5 and 2.5 meters per hour. Temperature and humidity conditions that may have a direct impact on the life cycle of the vectors were among the most important factors influencing the development of sand flies and their life cycle (16). In this research, the peak activity of sand flies was found to be exactly 18–18.5 °C and the average humidity was 80–86 percent. Two studies for India and Brazil have found similar results with respect to the direct effects of temperature and humidity on leishmaniasis vectors development (28–30).

## Conclusion

Sand flies were active from April to October (5–6 months on average), with on peak in July which is strongly influenced by temperature and humidity conditions. The vectors of visceral leishmaniasis in this study belonged to the Larrossius subgenus of phlebotomine sand flies, and two species of *Ph. kandelakii* and *Ph. perfilewi transcaucasicus*, had the highest activity in this endemic area. Serious actions must therefore be taken to manage vectors and avoid visceral leishmaniasis infections prior to the onset of activity (April) and, in particular, during peak activity (July).
